# Subjective and objective quality assessment of gastrointestinal endoscopy images: From manual operation to artificial intelligence

**DOI:** 10.3389/fnins.2022.1118087

**Published:** 2023-02-14

**Authors:** Peng Yuan, Ruxue Bai, Yan Yan, Shijie Li, Jing Wang, Changqi Cao, Qi Wu

**Affiliations:** ^1^The Key Laboratory of Carcinogenesis and Translational Research (Ministry of Education), Department of Endoscopy, Peking University Cancer Hospital and Institute, Beijing, China; ^2^Faculty of Information Technology, Beijing University of Technology, Beijing, China

**Keywords:** gastroscope images, motion blur, subjective and objective quality assessment, human visual system, semi-full combination subspace

## Abstract

Gastrointestinal endoscopy has been identified as an important tool for cancer diagnosis and therapy, particularly for treating patients with early gastric cancer (EGC). It is well known that the quality of gastroscope images is a prerequisite for achieving a high detection rate of gastrointestinal lesions. Owing to manual operation of gastroscope detection, in practice, it possibly introduces motion blur and produces low-quality gastroscope images during the imaging process. Hence, the quality assessment of gastroscope images is the key process in the detection of gastrointestinal endoscopy. In this study, we first present a novel gastroscope image motion blur (GIMB) database that includes 1,050 images generated by imposing 15 distortion levels of motion blur on 70 lossless images and the associated subjective scores produced with the manual operation of 15 viewers. Then, we design a new artificial intelligence (AI)-based gastroscope image quality evaluator (GIQE) that leverages the newly proposed semi-full combination subspace to learn multiple kinds of human visual system (HVS) inspired features for providing objective quality scores. The results of experiments conducted on the GIMB database confirm that the proposed GIQE showed more effective performance compared with its state-of-the-art peers.

## 1. Introduction

Gastric cancer (GC) is the major cause of cancer death worldwide (Chen et al., 2022). Recently, gastrointestinal endoscopy has been identified as an important tool for cancer diagnosis and therapy, particularly for treating patients with early gastric cancer (EGC) (Li Y.-D. et al., 2021). A proper application of endoscopy could identify and treat gastric lesions better. The main purpose of medical image processing and analysis is to facilitate physicians to conduct diagnosis and therapy (Cai et al., 2021; Xu et al., 2022). It is well known that the quality of gastroscope images is a prerequisite for achieving a high detection rate of gastrointestinal lesions (Liu et al., 2021). As gastroscope detection is operated manually, in practice, it possibly introduces motion blur and produces low-quality gastroscope images during the imaging process. These poor quality gastroscope images could lead to misdiagnosis, and thus patients must need a second examination that increases their pain one more time and even worse makes them miss the best time for treatment. Therefore, the image quality assessment (IQA) of gastroscope images is helpful to lead to more accurate and earlier detection, helping further in the development of image deblurring, enhancement, fusion, and denoising (Chen et al., 2021; Qin et al., 2021). To sum up, a good IQA method of gastroscope images is very important to determine lesions effectively.

In the field of image processing and computer vision, IQA is a crucial topic of research topic (Ye X. et al., 2020; Sun et al., 2021), including the subjective assessment and the objective assessment. The subjective assessment is widely perceived to be the most accurate IQA method because the measuring results of its image quality as the mean opinion score (MOS) are provided by human viewers. A few well-known and publicly available IQA databases with MOS or differential MOS (DMOS), such as Tampere Image Database 2013 (TID2013) (Ponomarenko et al., 2013), Categorical image quality (CSIQ) (Larson and Chandler, 2010), and Laboratory for Image and Video Engineering (LIVE) (Sheikh et al., 2006), pave the way for the development of the IQA. Over the past decade, many scholars have built several IQA databases for more practical purposes. For example, a contrast-changed image database (CCID2014) was included in Gu et al. (2015b) to enable a study on the perceptual quality of images with contrast changes. Two tone mapping image databases were presented in Kundu et al. (2017) and Gu et al. (2016b) to facilitate research on the quality of evaluation of tone-mapped images with a high dynamic range (HDR). The IQA database for super-resolved images was designed in Fei (2020) for assessing the visual quality of super-resolution images. However, well-known IQA databases are improper in the case of gastroscope images. Specifically, there is no specific subjective IQA database of gastroscope images. Because a gastroscope is placed inside the body, many types of distortion in these databases, such as impulse noise, brightness change, and Joint Photographic Experts Group (JPEG) compression, are not included in gastroscope images, but a motion blur usually exists. Up to now, to the best of our knowledge, there has been not a publicly available database for the quality assessment of gastroscope images, so it is highly necessary to establish an IQA database of distorted gastroscope images.

The MOS values are obtained experiments that include different individuals and circumstances, but which are improper for the real-time IQA of gastroscope images. The MOS is obtained in a labor-intensive and time-consuming process, and is thus of very low reusability. Another strategy to evaluate the quality of images which is highly demanding is to develop objective assessment methods toward matching the characteristics of a human vision system (HVS). Recently, objective IQA method have achieved good results. The typical objective IQA metrics are based on the full-reference (FR), where a “clean” gastroscope image is available. The “clean” gastroscope image is the ground truth in the case of gastroscope images distorted with motion blur. The visual signal-to-noise ratio (VSNR) Chandler and Hemami (2007) takes advantage of the supra- and near-threshold characteristics of human vision. The peak signal-to-noise ratio (PSNR) and mean-squared errors (MSEs) are the most popular and commonly used FR IQA techniques, but their correlation with perceived quality is not ideal. The most apparent distortion (MAD) Larson and Chandler (2010) method adaptively extracts visual features from the reference and distorted images using the log-Gabor filtering and Fourier transform. The structural similarity (SSIM) Wang et al. (2004) compares three visual aspects including contrast, luminance, and structure. Later on, many variants were proposed, based on the SSIM (Wang et al., 2003; Sampat et al., 2009; Wang and Li, 2010; Zhu et al., 2018).

The FR IQA methods also make use of many other cues or features, except for covariance, variance, and mean. Mutual information between the distorted and the lossless images is used to evaluate the quality of visual perception in the information fidelity criterion (IFC) (Sheikh et al., 2005) and its extended approach named the visual information fidelity (VIF) (Sheikh and Bovik, 2006). In addition, since it is known that image gradients contain many types of significant visual information, some IQA approaches extract the gradient features. In Zhang et al. (2011), the feature similarity (FSIM) was proposed to incorporate gradient magnitudes with phase congruency. In Liu et al. (2011), the gradient similarity (GSIM) was developed by combining gradient features with masking effect and distortion visibility. In Xue et al. (2013), the gradient magnitude similarity deviation (GMSD) takes advantage of a new pooling strategy that is the global variation of a local gradient similarity. Both the pooling weights and local features represent visual saliency of the image in the IQA (Zhang et al., 2014; Ye Y. et al., 2020). A few existing IQA models utilize the predictability as a feature. The different strategies of the unpredicted and predicted parts in an image are employed in Wu et al. (2012) to measure the internal generative mechanism (IGM) index.

However, the scope of application of FR IQA is constrained by the dependence of lossless images. In recent years, the no-reference (NR) IQA models have been emphatically developed to solve the problem of the original image not being available in many cases (Hu et al., 2021; Li T. et al., 2021; Pan et al., 2021; ur Rehman et al., 2022). In Gu et al. (2017c), the authors extracted 17 features including brightness, sharpness, contrast, and so on, and then achieved a predictive quality score by a regression model. In Gu et al. (2017d), the authors developed a novel blind IQA model for evaluating the perceptual quality of screen content images with big data learning. In Gu et al. (2014b), the authors proposed a new blind IQA model using the classical HVS features and the free energy feature based on the image processing and brain theory. In Gu et al. (2015c), the authors designed an NR sharpness IQA metric that is built using the analysis of autoregressive (AR) parameters. However, some distortion types, such as motion blur that may appear in the gastroscope images, are not considered in the majority of the existing IQA methods, so these off-the-shelf methods do not suit gastroscope images the best.

In this study, we attempt to construct a novel image database and a specific IQA metric of gastroscope images to identify and treat gastric lesions better. Because motion blur easily takes place in a gastroscope image during the imaging process, we focus mainly on how it affects the quality of a gastroscope image. First, we build a gastroscope image motion blur (GIMB) database that encompasses 70 source images from 27 categories of the upper endoscopy anatomy is built and 1,050 corresponding motion blurred images derived from five pixel levels for three different motion angles. We adopt the single stimulus (SS) method to gather subjective ratings. Then, we properly integrate the existing FR IQA methods (Wu et al., 2021) to design an artificial intelligence (AI)-based gastroscope image quality evaluator (GIQE). To define it more concretely, we learn multiple kinds of HVS inspired features from gastroscope motion blurred images by the newly proposed semi-full combination subspace. The results reveal that the proposed GIQE can achieve a superior performance relative to the state-of-the-art FR IQA metrics.

The remainder of this article is arranged as follows. In Section 2, the subjective assessment of gastroscope images and the establishment of the relevant GIMB database are introduced in detail. In Section 3, a detailed implementation of the proposed GIQE is presented. In Section 4, a comparison of the proposed GIQE with several mainstream FR IQA metrics is carried out using the GIMB database. In Section 5, some conclusions are finally drawn.

## 2. GIMB image database

In this section, we describe the proposed GIMB database. First, we introduce the formation and processing of source images. Then, the subjective methodology is leveraged to collect the MOS values from the viewers. Finally, the collected values of MOS are processed and analyzed.

### 2.1. The formation of source images

**Figure 1:**
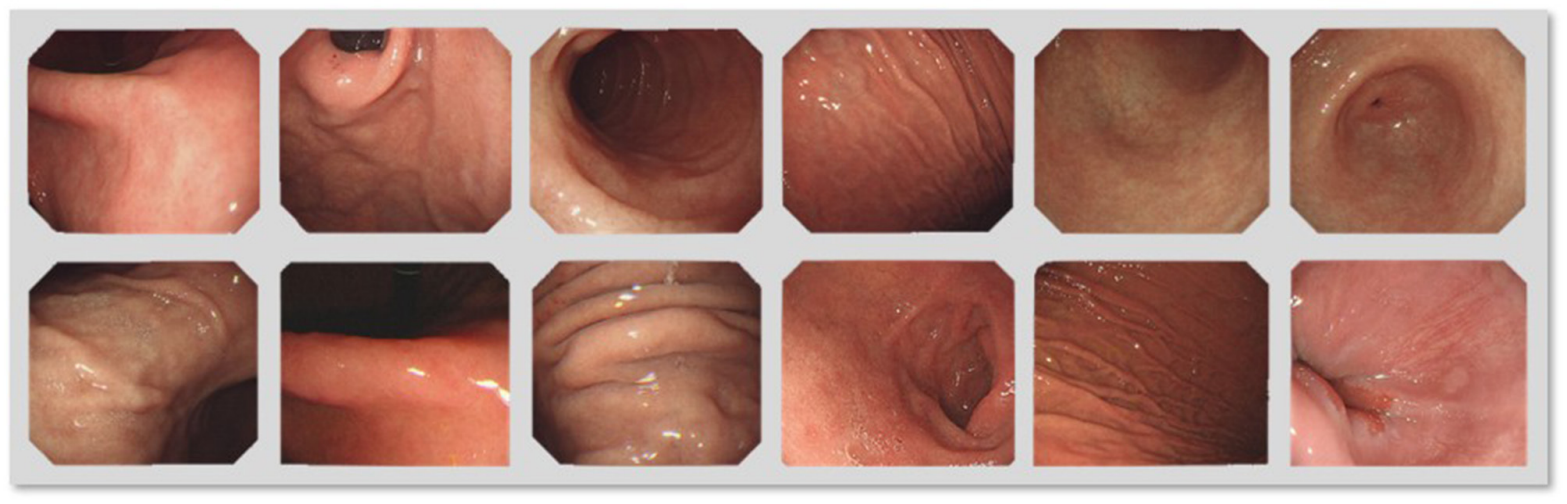
The source images in different gastrointestinal tract regions in the gastroscope image motion blur (GIMB) database.

It is nontrivial to select source images, because the content of source images has a strong effect on the IQA. According to the general theory, the source images ought to be undistorted, and their contents should be abundant and diverse. The GIMB database encompasses 70 source images that are taken from 27 categories of the upper endoscopy anatomy, such as the antrum anterior wall, the pharynx, the pylorus, and the fundus, as shown in Figure 1. In this study, the patients were examined by gastroscopy at the Peking University Cancer Hospital from June 2020 to December 2021. The Ethics Committee approved the study at the Peking University Cancer Hospital on 15 May 2020 (ethics board protocol number 2020KT60). The source images were captured by endoscopes such as GIF-H290, GIF-HQ290, GIF-H260 (Olympus, Japan), EG-760Z, EG-760R, EG-L600ZW7, EGL600WR7, and EG-580R7 (Fujifilm, Japan). Areas around gastroscope images contain information on indicators that does not contribute to the IQA and should therefore be removed. We cropped the source images into the same resolution of 1,075 × 935 to remove unnecessary information and obtain a higher processing level of IQA.

### 2.2. The processing of source images

From the perspective of an IQA database, the gastroscope blurred images are actually the images distorted by motion blur. The relative motion between the gastrointestinal tract regions and the probe during gastroscopy by artificial operation often leads to motion blur in gastroscope images. The motion blur is caused by the superposition of multiple images at different times. We set *x*_0_(*t*) and *y*_0_(*t*) as the motion components in *x* and *y*, and set *T* as the exposure time. The vague image adopted at time *t* is


(1) $$g(x,y)=\int_{0}^{T}f[x-x_{0}(t),y-y_{0}(t)]dt.$$

We suppose that the motion between the gastrointestinal tract regions and the probe is a kind of uniform rectilinear movement. During time *T*, the moving distances are represented by *a* and *b* in *x* and *y*:


(2) $$\{\begin{array}{c} x_{0}(t)=at/T\\ y_{0}(t)=bt/T \end{array}$$

Combining with Equations (1), (2), the probe moves *L* pixels with uniform speed in a straight line at θ angle in the *x*-*y* plane. The vague image is obtained by


(3) $$\begin{array}{l} \begin{array}{c} g(x,y)=\frac{1}{L}\sum_{i=0}^{L-1}f[x^{\prime }-i,y^{\prime }] \end{array} \end{array}$$

Where *x*′ = *x* cosθ + *y* sinθ and *y*′ = *y* cosθ − *x* sinθ. *i* ∈ {1, 2, 3,..., *L*-1} is an integer.

Therefore, we define the point spread function (PSF) of the motion blurred image in any direction by


(4) $$h(x,y)=\left\{ \begin{array}{l} 1/L\;y=x\tan\theta ,0\leq x\leq L\cos\theta \\ 0\;y\neq (x\tan\theta ),-\infty <x<\infty \end{array}\right.$$

Two important parameters include the direction of the motion blur θ and the distance from where the pixels *L* have blurred.

**Figure 2:**
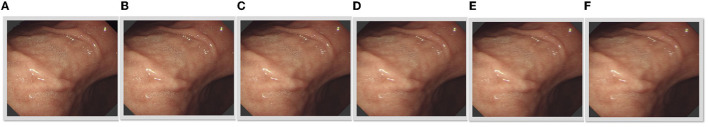
The five motion blur levels of a lossless image **(A)**. **(B–E)** Are the motion blurred image with five levels corresponding to lossless image. **(D)** Shows the boundary between available **(B, C)** and unavailable **(E, F)**.

To obtain motion blurred images, we processed source images using the built-in function of MATLAB application. To be more specific, we used two key parameters, *L* and θ, of motion blur aforementioned to process each lossless image. We set the direction of the motion blur to be at three different motion angles θ = {30°, 60°, and 90°}. Because gastroscope images are different from natural images, their rotations have no impact on the diagnosis of doctors. In addition, we set the motion distance to be five pixel levels, that is, *L* = {5, 10, 15, 20, 25}, which directly affect the performance of the IQA and the detection rate of gastric lesions. Figure 2 shows five motion blur levels of a lossless image. For the five motion blur grades, doctors agree that *L* = {5, 10} is useful for diagnosis, while *L* = {5, 10} corresponds to poor quality gastroscope images, potentially contributing to the misdiagnosis. Moreover, *L* = 15 is the boundary between the availability and the unavailability as confirmed by most of the physicians. On this basis, we generated 15 motion blurred images from each source image. Overall, the proposed GIMB database contains 70 lossless images and 1,050 distorted images.

### 2.3. Subjective methodology

Subjective methodology is an important procedure in creating an IQA database, yet it is very labor-intensive and time-consuming. In the following, we present the subjective test method, subject, environment, and the apparatus.

#### 2.3.1. Method

The methodology for the subjective assessment of the quality of television pictures. Recommendation ITU-R BT.500-13 (Ritur, 2002) has defined several subjective test methods that include SS, double-stimulus impairment scale (DSIS), and paired comparison. In this study, we used the SS method to conduct the subjective experiment. The order of all test images on the database was randomized to minimize the impact of subjects' memories on MOS. The subjects were asked to score the quality of each gastroscope image from 1 to 5, according to their overall sensation to these images. The test was divided into four subsessions, each of which lasted <20 min. A subsession includes 18 min for scoring and 2 min for training, and the interval for each subsession lasts 5 min.

#### 2.3.2. Subject

**Figure 3:**
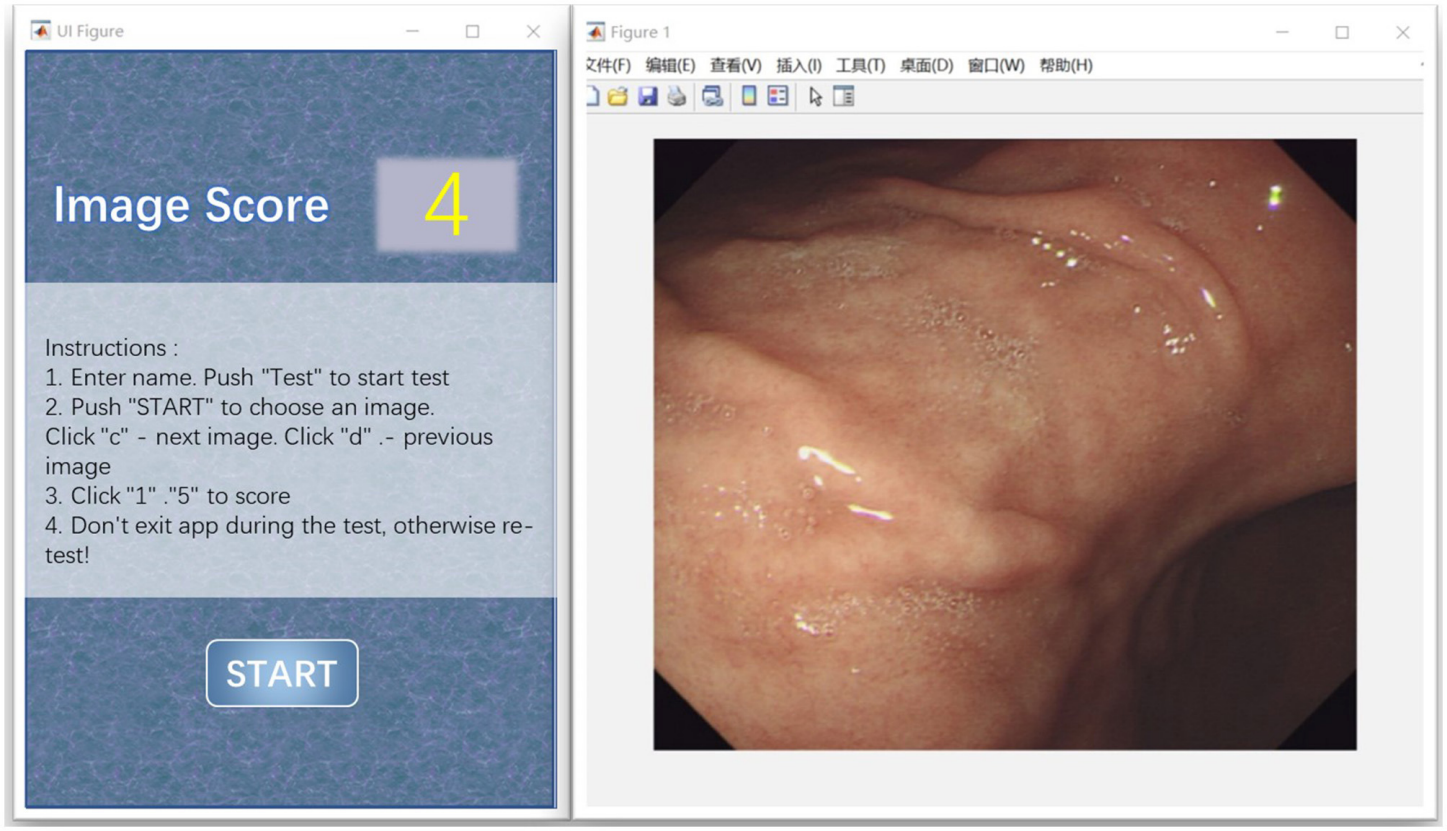
The interface window applied in the subjective assessment.

This subjective experiment involves experienced and inexperienced viewers, most of whom are physicians and postgraduates from the medical specialty. The inexperienced subjects are ignorant about distorted images and the corresponding terminology. Specific visual acuity tests including vision and color are not needed since the gastroscope image is a classical two-dimensional (2D) image. The subjects could wear their own glasses with suitable degree they wear every day. Before the test, we gave the viewers oral and written instructions, as specified in the International Telecommunication Union Telecommunication Standardization Sector (ITU-T) Recommendation. P.910. In the training phase, each subject is shown different pixel levels of motion blur, from the lowest to the highest as given in Figure 3, and familiar with the scoring procedure. The images used in the training stage and testing stage are different.

#### 2.3.3. Environment

To achieve reliable scoring results, we conducted the test in a fixed and controlled environment. Specifically, all the viewers were asked to perform their assessment in an indoor environment without any background light (Huang et al., 2021; Shi et al., 2021). We chose the suitable ambient luminance (Gu et al., 2015a; Yu and Akita, 2020). In the training phase, the viewing distance was set to approximately three times the image height. To get more precise scores, the viewers were able to modify the distance between the monitor and themselves slightly after a round testing.

#### 2.3.4. Apparatus

**Figure 4:**
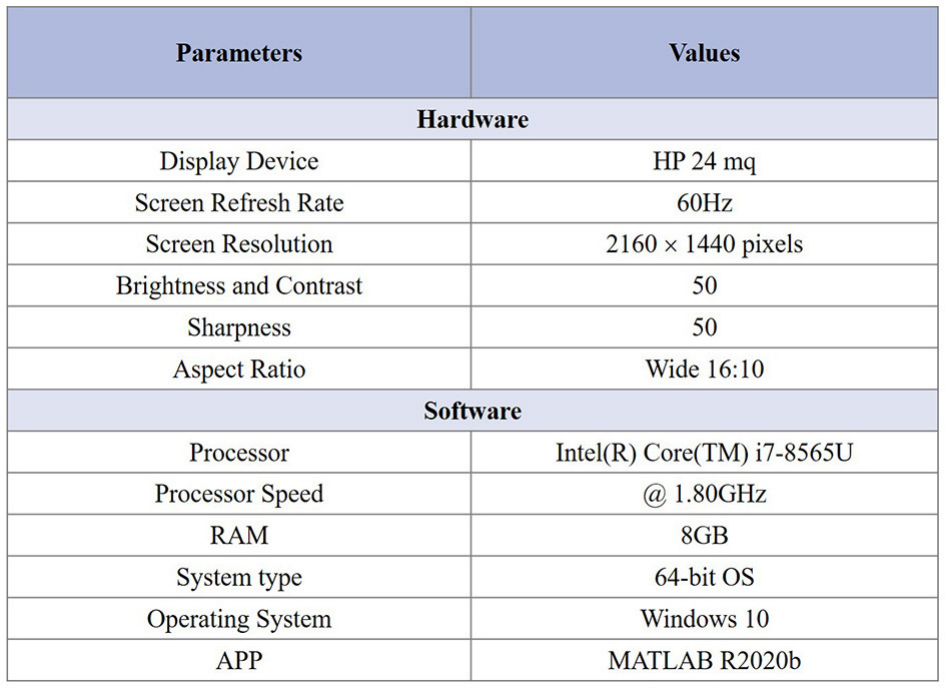
The characteristics of the display device and system are used in the experiment.

Two interface windows are shown simultaneously in MATLAB application and are applied to subjective assessment, as illustrated in Figure 3. The left window is used to score, while the right window is used to show the gastroscope motion gastroscope motion blurred image. The right window can be controlled by subjects during the test. The subjects can control which images should be shown in this window by pressing the key “c” or “d” on the keyboard. According to the psychovisual evaluation, we found that the viewers can make their decisions much more precisely and quickly by flipping the images at exactly the same position. Information about the psychovisual evaluation in detail is given in online materials section in Zhou et al. (2018). During scoring, the subjects were asked to give their scores as early as possible and were guided to click the button “1,” “2,” “3,” “4,” or “5” on the window, indicating their grading of motion blur from the lowest to the highest. The characteristics of the display device and system used in the experiment are described briefly in Figure 4. We saved the final scores of all the gastroscope images given by all the viewers after the subjective test for further analysis.

### 2.4. Scores processing and analysis

**Figure 5:**
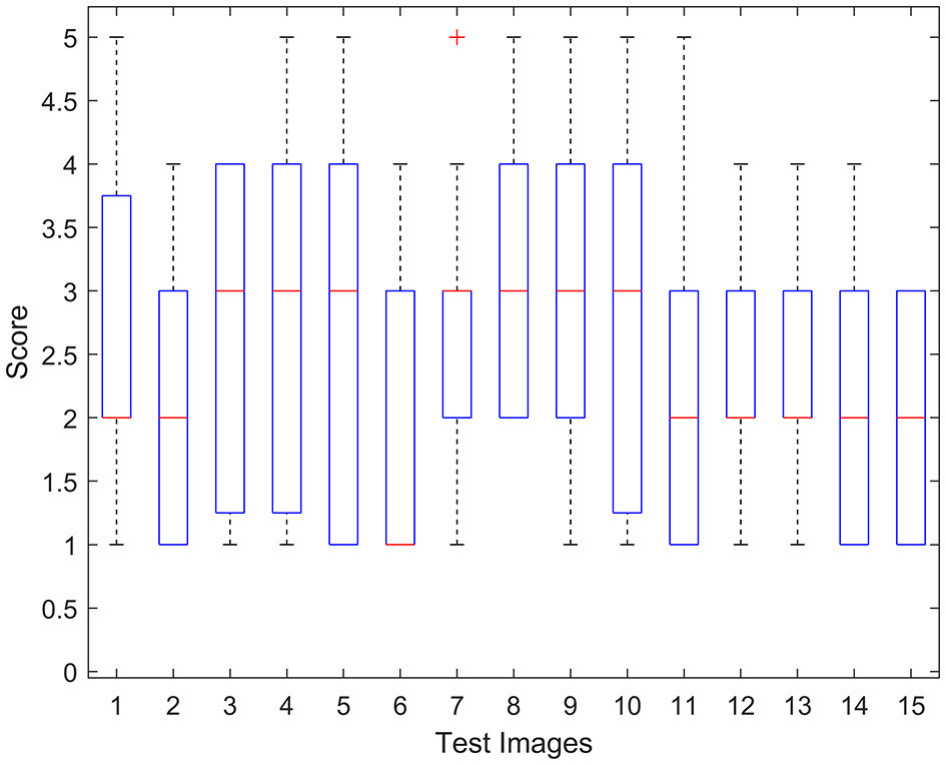
Box plot of the subjects' scores for 15 motion blurred images of a lossless image. On each image box, the central red line is the median score, the edges of the box are first quartile and third quartile, and the outliers are marked by a red cross individually.

According to the subjective test aforementioned, we gathered the viewers' scores to be processed and analyzed as follows:

First, we analyzed 15 motion blurred images of a lossless image using the box plot to study the influence of inattentive subjects on an individual observer's rating. The box plot (i.e., box and whisker diagram) is used to analyze the distribution of data on the basis of five indicators, including minimum, maximum, median, and the 25th and 75th percentiles. The range of the first and third quartiles is obvious, and there are a few points that are outliers, as shown in Figure 5. These results indicate that it is worthwhile to analyze the subjective score of an individual participant. Thus, we invited an experienced physician to screen the outliers.

Then, we processed all the values within the normal range after elimination. We assigned *m*_*ij*_ as the raw subjective score obtained from the viewer's *i* evaluation of the gastroscope motion blurred image *I*_*j*_, where *i* = {1, 2, 3, …, 15}, *j* = {1, 2, 3, …, *N*}, *N* < 1, 050. For a *j*^*th*^ image, the MOS value is calculated by the formula as follows:


(5) $$\begin{array}{l} \begin{array}{c} {\text{MOS}}_{j}=\sum_{i=1}^{M}\frac{m_{ij}}{M} \end{array} \end{array}$$

**Figure 6:**
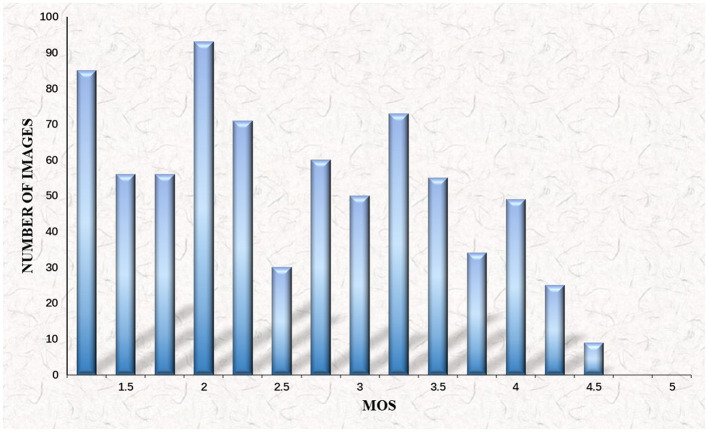
Histogram of mean opinion score (MOS) values for gastroscope motion blurred images.

Where *M* = 15 represents the number of subjects. We draw on the distribution histogram of the MOS to display the viewers' MOS scores as illustrated in Figure 6. An important observation indicates that the MOS scores of most distorted gastroscope images are only around 3.5 in comparison. Hence, motion blur influences the original gastroscope images considerably, which leads to a misdiagnosis of GC.

## 3. Proposed IQA metric

The existing FR and NR IQA models designed for a specific distortion category and an application scenario perform well, but are not suitable for gastroscope images. We explore an IQA metric for gastroscope motion blurred images using the semi-full combination subspace. Specifically, the image quality evaluation method of a gastroscope image is carried out in three steps.

### 3.1. The first step

We extract five features of gastroscope images since the processing of IQA is to learn multiple kinds of HVS inspired features. We then fuse these features to train a regression module.

#### 3.1.1. The low-level similarity feature

The phase congruency (PC) principle postulates that the Fourier transform phase contains maximal perceptual information, which helps the HVS to detect and identify features, according to the psychophysical and physiological evidence. Hence, we compute the feature *F*_*PC*_:


(6) $$\begin{array}{l} F_{PC}=\underset{\bar{\phi }\left(i\right)\in \left[0,2\pi \right]}{\max}\left\{\frac{\sum_{\mu }|F_{\mu }|\cos\left[\phi_{\mu }\left(i\right)-\bar{\phi }\left(i\right)\right]}{\sum_{\mu }|F_{\mu }|}\right\} \end{array}$$

Where |*F*_μ_| is the amplitude of an image and ϕ_μ_(*i*) represents the phase of *F*_μ_ at pixel *i* on the scale μ.

The gradient magnitude (GM) is a very classical and valid feature for improving the IQA performance. We employ the Scharr operator defined as $GM=\sqrt{GM_{x}^{2}+GM_{y}^{2}}$, where *GM*_*x*_ and *GM*_*y*_ are the partial derivatives along *x* and *y* axis directions. This GM is regarded as


(7) $$\begin{array}{l} F_{GM}=E\left[\frac{2GM\left(I_{d}\right)\cdot GM\left(I_{r}\right)+A_{1}}{GM{\left(I_{d}\right)}^{2}+GM{\left(I_{p}\right)}^{2}+A_{1}}\right] \end{array}$$

Where *I*_*r*_ is the reference image. *I*_*d*_ and *I*_*p*_ represent the distorted and predicted versions of *I*_*r*_, respectively. *A*_1_ is a fixed positive constant. Many recently proposed IQA algorithms have proven that PC and GM are very valid, since the HVS is very sensitive to them.

We then combine *f*_*PC*_ with *f*_*GM*_ to obtain the similarity feature *F*_*L*_, which is defined by


(8) $$\begin{array}{l} F_{L}=F_{PC}^{\alpha }\cdot F_{GM}^{\beta } \end{array}$$

Where parameters α and β are applied to change the importance of *f*_*GM*_ and *f*_*PC*_. Since the visual cortex is very sensitive to PC features, we use *F*_*PC*_ as a weight value to extract the low-level similarity feature *F*_*LSF*_ as the first feature:


(9) $$\begin{array}{l} F_{LSF}=\frac{F_{L}\cdot F_{PC}}{F_{PC}} \end{array}$$

#### 3.1.2. The visual saliency feature

Visual saliency (VS) areas of an image attract maximum attention of the HVS. We fuse VS, GM, and chrominance features to obtain the visual saliency feature (VSF) of images for IQA tasks. We extract VS maps of original and lossless images by a specific VS model. The similarity between them is defined as:


(10) $$\begin{array}{l} F_{VS}=E\left[\frac{2VS\left(I_{d}\right)\cdot VS\left(I_{r}\right)+A_{2}}{VS{\left(I_{d}\right)}^{2}+VS{\left(I_{p}\right)}^{2}+A_{2}}\right] \end{array}$$

The similarity between the chrominance featue components is simply defined as:


(11) $$\begin{array}{l} F_{C}=E\left[\frac{2M\left(I_{d}\right)\cdot M\left(I_{r}\right)+A_{2}}{M{\left(I_{d}\right)}^{2}+M{\left(I_{p}\right)}^{2}+A_{2}}\right]\cdot E\left[\frac{2N\left(I_{d}\right)\cdot N\left(I_{r}\right)+A_{2}}{N{\left(I_{d}\right)}^{2}+N{\left(I_{p}\right)}^{2}+A_{2}}\right] \end{array}$$

Where parameters *M* and *N* are the numbers of channels. *A*_2_ is another fixed positive constant.

We define *F*_*VSF*_ as the second feature:


(12) $$\begin{array}{l} F_{VSF}=F_{VS}\cdot F_{GM}^{\alpha }\cdot F_{C}^{\beta } \end{array}$$

Where two parameters α and β are used to adjust the relative importance of VS, GM, and chrominance features.

#### 3.1.3. The log-gabor filter

The log-Gabor filter (LGF) has strong robustness for brightness and contrast changes of images, and it has been widely used to extract local features and texture analysis in computer vision. We use a log-Gabor filter bank to decompose the source and lossless images into a set of subbands. The subband's features are obtained by the inverse density functional theory (DFT) of the images' DFT with the following multiplying 2D frequency response as the third feature:


(13) $$\begin{array}{l} F_{LGF}\left(f_{r},f_{\theta }\right)=\exp\left[-\frac{{\left(logf_{r}/f_{rs}\right)}^{2}}{2{\left(log\sigma_{s}/f_{rs}\right)}^{2}}\right]\times \exp\left[-\frac{{\left(f_{\theta }-\mu_{0}\right)}^{2}}{2\sigma_{0}^{2}}\right] \end{array}$$

Where *F*_*LGF*_(*f*_*r*_, *f*_θ_) is a log-Gabor filter by two indexes, which are the normalized radial frequency $f_{r}=\sqrt{{\left(\mu /M/2\right)}^{2}+{\left(\mu /N/2\right)}^{2}}$, and the angle of orientation *f*_θ_ = arctan(υ/μ). The parameter *f*_*rs*_ is the normalized center frequency of the scale, and the bandwidth of the filter is determined by σ_*s*_/*f*_*rs*_. The parameters μ_0_ and σ_0_ denote the orientation and angular spread of the filter, respectively. The parameters *f*_*rs*_, σ_*s*_, μ_0_, and σ_0_ can be determined by the corresponding evaluation derived from the HVS, since it is known that the log-Gabor filter approximates cortical responses in the primary visual cortex.

#### 3.1.4. The mutual information feature

The mutual information represents the amount of feature information that we can extract from the HVS output. For the source or lossless images, we define the mutual information to be the fourth feature by


(14) $$\begin{array}{l} F_{MIF}=\frac{1}{2}\overset{N}{\underset{i=1}{\sum }}log_{2}\left(\frac{x_{i}^{2}|B_{U}+\epsilon_{m}^{2}\text{I}|}{\left|\epsilon_{m}^{2}\text{I}\right|}\right) \end{array}$$

Where *x* = {*x*_*i*_ : *i* ∈ *I*} is an RF of positive scalars and *U* is a Gaussian vector RF with mean zero and covariance *B*_*U*_. $B_{U}=Q\text{Λ}Q^{T}$ is symmetric. The parameter *Q* is an orthonormal matrix and Λ is a diagonal matrix. $\epsilon_{m}^{2}$ represents the variance of the visual noise. The RFs *M* and *N* are supposed to be independent of *U* and $B_{M}=\epsilon_{m}^{2}$ I. |·| denotes the determinant.

#### 3.1.5. The novelty structural feature

The HVS is sensitive to structural distortion since natural images are highly structured. The structural feature of an image represents the structure of objects in the scene, different from the contrast and luminance. For example, as regards SSIM, Wang et al. (2004) calculate the differences in a few features (i.e., contrast, structural, and luminance) between *I*_*r*_ and *I*_*d*_. Multiscale structural similarity (MS-SSIM) (Wang et al., 2003) mainly incorporates contrast and structural similarities that are more effective than the luminance similarity in SSIM. We compute the contrast similarity:


(15) $$\begin{array}{l} F_{SF}=E\left[\frac{2\eta_{\left(I_{d}\right)}\eta_{\left(I_{r}\right)}+A_{3}}{\eta_{\left(I_{d}\right)}^{2}+\eta_{\left(I_{r}\right)}^{2}+A_{3}}\right] \end{array}$$

Where η_(_*I*__*d*_)_ and η_(_*I*__*r*_)_ are the gradient values for the central pixel of images *I*_*d*_ and *I*_*r*_, respectively. *A*_1_, *A*_2_, and *A*_3_ are all fixed. *E*(·) represents the expectation or the mean value. In the pixel version, we define *F*_*NFS*_ to be the fifth feature:


(16) $$\begin{array}{l} F_{NSF}\left(i,j\right)=\frac{2\left(1-R\right)+K}{1+{\left(1-R\right)}^{2}+K} \end{array}$$

Where $R=\frac{\left|f_{SF}\left(i\right)-f_{SF}\left(j\right)\right|}{\text{max}\left[f_{SF}\left(i\right),f_{SF}\left(j\right)\right]}$ and $K=A_{3}/\text{max}{\left[f_{SF}\left(i\right),f_{SF}\left(j\right)\right]}^{2}$ of image blocks *i* and *j*.

### 3.2. The second step

Inspired by Gu et al. (2020), we propose a semi-full combination subspace method, which is an elaborate integration of bootstrapping and aggregation applied to environmental factors. The semi-full combination subspace exerts bootstrapping on the input features. A high-dimensional feature vector or a small number of training samples is very likely to lead to an overfitting. Specifically, directly using all of the aforementioned five features is not always superior to the situation of using only a few of them. To address this issue, a new subset composed of a segment of the features is generated, which decreases the conformity between the length of the feature vector and the size of the training sample. Using the new semi-full combination subspace, we can obtain a component learner. By applying the aforementioned process to the feature space repeatedly through the feature selection, we can build multiple component learners with diversity of the environmental factors.

### 3.3. The third step

**Figure 7:**
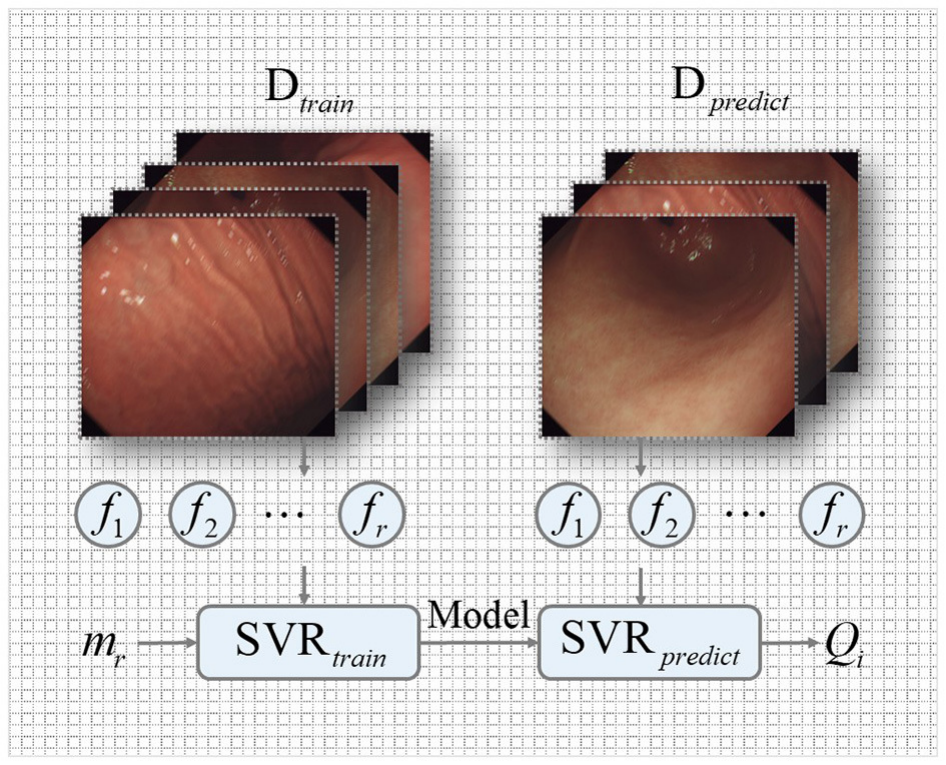
The implementation of an efficient regression engine.

We use the semi-full combination subspace of five features to attain a single direct visual quality of gastroscope images. An efficient regression engine, namely support vector regression (SVR) (Mittal et al., 2012), is used to reliably transform the semi-full combination subspace into a single objective quality score. Concretely, we implement the SVR by the radial basis function (RBF) kernel (Mittal et al., 2012) included in the LibSVM package, as shown in Figure 7.

#### 3.3.1. SVR training

We train an SVR to learn a regression model using the GIMB database. This database contains a number of different gastrointestinal tract regions and motion blur levels. To train our proposed model, we split the GIMB into 40% data for testing and 60% data for training. The SVR has significant advantages of high efficiency and flexibility.

We consider the GIMB training database *T* = {(*f*_1_, *m*_1_), (*f*_2_, *m*_2_), …, (*f*_*r*_, *m*_*r*_)}, where *f*_*r*_ and *m*_*r*_, *r* = {1, …, *N*}. *f*_*r*_ indicates a feature vector of *f*_1_ − *f*_5_ of the *r*th training image. The training labels *m*_*r*_ are subjective MOSs. We express the linear soft-margin SVR as


(17) $$\begin{array}{l} \underset{w,b,\xi_{r},\hat{\xi_{r}}}{\min}\;\frac{1}{2}\|W{\|}^{2}+\;H\sum_{i=1}^{N}\left(\xi_{r},\hat{\xi_{r}}\right)\\ s.t.\;mode-m_{r}\leq \varepsilon -\xi_{r},\\ \;m_{r}-mode\leq \varepsilon +\hat{\xi_{r}},\\ \;\xi_{r}\geq 0,\hat{\xi_{r}}\geq 0,r=1,2,\ldots ,N \end{array}$$

Where we set kernel function *K*(*f*_*r*_, *f*_*i*_) to be the RBF kernel defined by


(18) $$K(f_{r},f_{i})\;=\varphi {\left(f_{r}\right)}^{T}\varphi (f_{i})=exp(-k\|x_{r}-x_{i}{\|}^{2})$$

By training the SVR on the GIMB database, we want to determine the optimal parameters *H*, ε, and *k* to obtain a fixed regression model, which is defined as


(19) $$\begin{array}{l} model={\text{SVR}}_{train}\left(f_{r},m_{r},D_{train}\right) \end{array}$$

Where *D*_*train*_ is the training set. Five features are extracted to create a model named the gastroscope image quality evaluator (GIQE).

#### 3.3.2. SVR prediction

Finally, the performance of the proposed GIQE metric is verified on testing the GIMB database with the obtained model. The perceived quality score *Q*_*j*_ of GIQE for gastroscope images is computed by


(20) $$\begin{array}{l} Q_{i}={\text{SVR}}_{predict}\left(f_{j},model,D_{predict}\right) \end{array}$$

Where *D*_*predict*_ is the testing set.

## 4. Comparison of objective quality assessment metrics

In this section, we investigate whether several existing FR IQA models can evaluate the quality of gastroscopic motion blurred images effectively. There are 20 traditional and mainstream FR IQA methods. Four commonly used performance indicators are adopted to compute the correlation between each MOS and FR IQA metric.

### 4.1. Objective quality assessment models

We introduce some categories of FR IQA algorithms as follows:

- Analysis of distortion distribution-based SSIM (ADD-SSIM): Gu et al. (2016a) propose a high-performance fusion model based on the SSIM by analyzing the distortion distribution influenced by the image content and distortion.
- MAD: Larson and Chandler (2010) evaluates the perceived quality of low- and high-quality images using two different strategies respectively.
- Visual signal-to-noise ratio (VSNR): Chandler and Hemami (2007) uses image features to estimate the image quality in the wavelet domain, visual masking, near-threshold, and supra-threshold properties.
- Analysis of distortion distribution GSIM (ADD-GSIM): Gu et al. (2016a) incorporate the frequency variation, distortion intensity, histogram changes, and distortion position distributions to infer the image quality.
- IFC: Sheikh et al. (2005) use the natural scene statistics captured by sophisticated models to propose a novel information fidelity criterion (IFC).
- VIF: Sheikh and Bovik (2006) considers it an information fidelity problem to quantify the loss of distorted images and explore the correlation between visual quality and images.
- Visual information fidelity in pixel domain (VIFP): Sheikh and Bovik (2006) develops a novel version of VIF in the pixel domain to reduce computational complexity.
- IGM: Wu et al. (2012) control the process of cognition according to the basic hypothesis of the free-energy-based brain theory.
- Local-tuned-global model (LTG): Gu et al. (2014a) assume that the HVS draws on the prominent local distortion and global quality degradation to characterize the image quality.
- Noise quality measure (NQM): Damera-Venkata et al. (2000) combine the local luminance mean, contrast pyramid of Peli, contrast sensitivity, contrast mask effects, and contrast interaction in spatial-frequency domain.
- Reduced-reference image quality metric for contrast change (RIQMC): Gu et al. (2016a) design a novel pooling module by the analysis of distortion intensity, distortion position, histogram changes, and frequency changes to infer an overall quality measurement.
- Structural variation-based quality index (SVQI): Gu et al. (2017b) evaluate the perceived quality of image based on the analysis of global and local structural variations on account of transmission, compression, etc.
- Perceptual similarity (PSIM): Gu et al. (2017a) take into account the similarities of GM at two scales and color information, and an effective fusion based on perception.
- GMSD: Xue et al. (2013) explore a novel fusion strategy according to the pixelwise gradient magnitude similarity (GMS) between the lossless image and the corresponding distorted image.
- FSIM and Feature similarity in color domain (FSIMC): Zhang et al. (2011) compute the PC and the similarity of GM between the lossless image and the distorted image.
- GSIM: Liu et al. (2011) combine gradient features with visual distortion and masking effect.
- Visual saliency-induced index (VSI): Zhang et al. (2014) skillfully combine the GM variations and vision saliency to perceive the image quality.

### 4.2. Performance of the objective quality assessment models

After introducing the aforementioned objective quality assessment models, we first map the objective predictions of the IQA models by the five-parameter logistic function:


(21) $$\begin{array}{l} \begin{array}{c} Q\left(x\right)=\beta_{1}\left(\frac{1}{2}-\frac{1}{1+e^{\beta_{2}\cdot \left(z-\beta_{3}\right)}}\right)+\beta_{4}\cdot z+\beta_{5} \end{array} \end{array}$$

Where *x* and *Q*(*x*) represent the input scores and the mapped scores, respectively. *z* is the predicted score of the IQA. β_*i*_(*i* = 1, 2, …, 5) are variable parameters that have to be defined in the fitting process.

Then, we draw on four statistical indicators, as detailed in Zhang et al. (2014), to compare the consistency of the predicted ratings from subjective MOSs and objective IQA models. The four indicators represent different meanings and evaluate the predicted performance in different ways. First, Pearson's linear correlation coefficient (PLCC) points out the accuracy by computing correlation of the subjective and objective scores. Second, Spearman's rank-order correlation coefficient (SROCC) reflects the predicted monotonicity of IQA, which does not dependent on any monotone nonlinear mapping between the objective scores and MOSs. Third, Kendall's rank-order correlation coefficient (KROCC) is a nonparametric rank correlation metric to measure the matching between the original scores and the converted objective ones. The last root mean-squared error (RMSE) indicates the predicted consistency, which is defined as the energy between two data sets. For the four indicators aforementioned, a superior IQA model means the values of PLCC, SROCC, and KROCC are close to 1, while the value of RMSE is close to 0. Table 1 lists the performance of 20 FR IQA models on PLCC, SROCC, KROCC, and RMSE. The best performing objective methods are highlighted in boldface in each column.

**Table 1:** Performance comparison of the proposed gastroscope image quality evaluator and the existing full-reference image quality assessment (FR IQA) metrics on the gastroscope image motion blur (GIMB) database.

	**PLCC**	**SROCC**	**KROCC**	**RMSE**
PSNR	0.6463	0.5703	0.4042	0.6966
MSE	0.4379	0.5833	0.4139	0.8207
SSIM	0.5831	0.5313	0.3730	0.7416
ADD-SSIM	0.7608	0.7022	0.5099	0.5924
MS-SSIM	0.7826	0.7248	0.5493	0.5683
FSIM	0.8571	0.8325	0.6343	0.4702
FSIMC	0.8568	0.8319	0.6336	0.4708
PSIM	0.7335	0.6780	0.4904	0.6205
MAD	0.8585	0.8412	0.6404	0.4682
VSNR	0.6027	0.5312	0.3707	0.7284
GMSD	0.7813	0.7090	0.5266	0.5697
GSIM	0.8486	0.8205	0.6237	0.4829
ADD-GSIM	0.7657	0.7026	0.5 135	0.5871
VIF	0.8392	0.8285	0.6329	0.4964
VIFP	0.8575	0.8269	0.6319	0.4697
IGM	0.7836	0.7111	0.5263	0.5671
LTG	0.7690	0.7038	0.522 1	0.5836
NQM	0.8533	0.8376	0.6375	0.4760
VSI	0.8316	0.8027	0.6050	0.5070
IFC	0.8630	0.8501	0.6578	0.4612
GIQE	**0.8883**	**0.8849**	**0.6988**	**0.7766**

The best results of each indicator are highlighted in bold.

We compared the performance of 20 commonly used FR IQA models for gastroscope motion blurred images. From Table 1, we derive some important conclusions as follows:

(1) The top two IQA models are highlighted in different bold colors to compare our method with those of other competitors straightaway. It is obvious that the proposed GIQE model, whose PLCC, SROCC, KROCC, and RMSE reach 0.8883, 0.8849, 0.6988, and 0.7766, respectively, shows a better performance than the existing FR IQA models.

Specifically, we concentrate only on the PLCC indicator, and similar conclusions can be drawn from the other three indicators. IFC is the second best performing model, achieving 0.8630 on PLCC. Compared with the IFC, the performance of the proposed IQA metric GIQE has improved by 2.9%. The performance gains of the proposed GIQE models are 13.5 and 16.7% higher than those of MS-SSIM and ADD-SSIM, respectively.

(2) We can see that a few aforementioned FR IQA models do not exhibit a remarkably high correlation with subjective quality. For example, the performance of PSNR and VSNR for the gastroscope motion blurred images is low. It means that the assessment model is not suitable for the study of gastroscope images. Since the gastroscope is placed inside the body, the images it produces do not contain most types of distortion found in natural images, such as pulse noise, brightness changes, and JPEG. It causes the PSNR to be inferior to the traditional successful methods for natural images.

(3) We study the performance of the SSIM and SSIM-based FR IQA models for gastroscope motion blurred images. The performance of all SSIM-based IQA models has showed an improvement compared with that of SSIM, indicating that they can promote the analysis of motion blurred distortion in gastroscope images. Both ADD-SSIM and MS-SSIM analyze the influence of motion blurred distortion on image's structure. MS-SSIM performs the best among these SSIM-based IQA methods and it obtains values 0.7826, 0.7248, 0.5493, and 0.5683 of PLCC, SROCC, KROCC, and RMSE, respectively. Yet, SSIM obtains values 0.5831, 0.5313, 0.3730, and 0.7416 of PLCC, SROCC, KROCC, and RMSE, respectively, which is the worst performing model among all FR IQA tested methods. It shows that the motion blurred distortion caused by the superposition of multiple images at different times has a great effect on the structure of images.

(4) We find that the methods based on the image gradients, such as FSIM, FSIMC, and GSIM, achieve a high performance in terms of these traditional FR IQA metrics, as image gradients are significant in gastroscope images. VIF, VIFP, and VSI metrics achieve superior performance than most of the tested FR IQAs, which indicate that the features extracted by VIF, VIFP, and VSI metrics are less affected by the motion blur. It brought to light the fact that the visual saliency features of VIF, VIFP, and VSI models are useful for assessing the quality of gastroscope motion blurred images. In addition, the saliency models used in VIF, VIFP, and VSI models are not specially devised for motion blurred images.

(5) Among the existing FR IQA methods, IFC shows the best performance, which achieves values 0.8630, 0.8501, 0.6578, and 0.4612 of PLCC, SROCC, KROCC, and RMSE, respectively. This observation indicates that IFC has the highest correlation with the perceptual scores for gastroscope images. However, the performance of IFC is far from satisfactory. All the existing FR IQA methods do not take into consideration the distorion-specific category of the gastroscope image. The objective algorithm for gastroscope images needs to be studied further.

**Figure 8:**
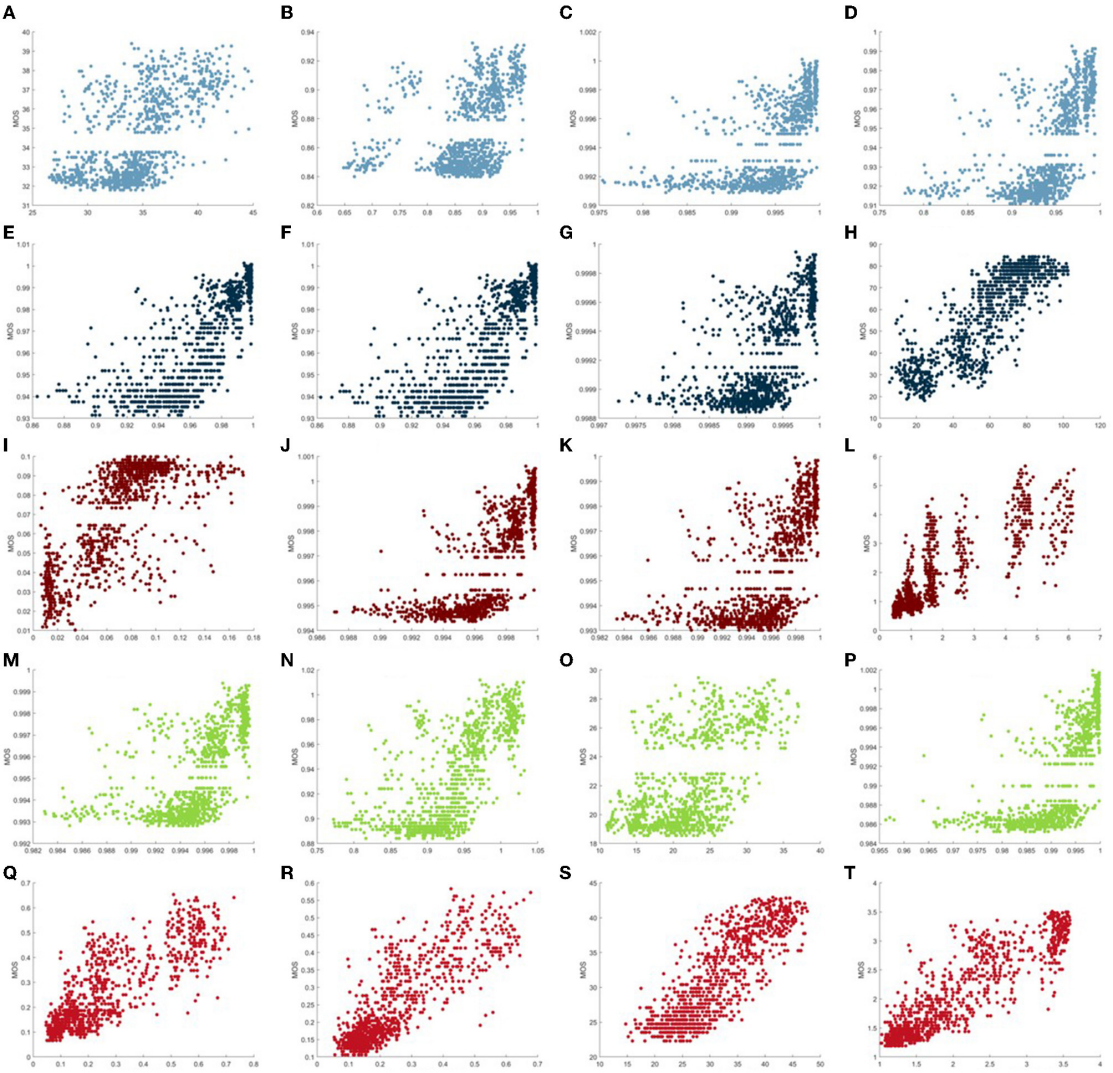
Scatter plots of mean opinion scores (MOS) vs. the proposed **(T)** gastroscope image quality evaluator (GIQE) and traditional Full-Reference Image Quality Assessment (IQA) models [**(A)** peak signal-to-noise ratio (PSNR), **(B)** structural similarity (SSIM), **(C)** analysis of distortion distribution-based SSIM (ADD-SSIM), **(D)** multi-scale structural similarity (MS-SSIM), **(E)** feature similarity (FSIM), **(F)** feature similarity in color domain (FSIMC), **(G)** perceptual similarity (PSIM), **(H)** most apparent distortion (MAD), **(I)** gradient magnitude similarity deviation (GMSD), **(J)** gradient similarity (GSIM), **(K)** analysis of distortion distribution GSIM (ADD-GSIM), **(L)** information fidelity criterion (IFC), **(M)** local-tuned-global model (LTG), **(N)** internal generative mechanism (IGM), **(O)** visual signal-to-noise ratio (VSNR), **(P)** visual saliency-induced index (VSI), **(Q)** visual information fidelity (VIF), **(R)** visual information fidelity in pixel domain (VIFP), and **(S)** noise quality measure (NQM)].

The scatter plot is a common manifestation of comparison in the IQA study, which can show some direct-viewing illustrations of different IQA models. In Figure 8, we provide the scatter plots of MOS vs. 20 existing objective FR IQA methods tested on the proposed GIMB database. These representative models are composed of PSNR, SSIM, ADD-SIMM, MS-SSIM, FSIM, FSIMC, PSIM, MAD, VSNR, GMSD, GSIM, ADD-GSIM, IFC, VIF, VIFP, VSI, VIF, VIFP, IGM, LTG, and NQM. It can be seen that the sample points of IFC, MAD, NQM, and VIF present better convergence and linearity, which illustrates that these models can deliver more consistent results between the objective scores and the subjective scores. From Figure 8, we find that the proposed GIQE method (i.e., the last scatter plot) is more robust and shows a better performance with regard to correlation than the existing FR IQA models (including IFC, MAD, NQM, and VIF). Particularly, the sample points of the proposed GIQE metric are quite close to the centerline, whereas those of the majority of other tested FR IQA models are far from the centerline. According to this, we assume that the proposed GIQE method demonstrates higher consistency in prediction performance.

## 5. Conclusions

In this study, we have investigated comprehensively a significant quality assessment problem of gastroscope motion blurred images in EGC diagnosis and therapy systems. We built a carefully devised GIMB database to facilitate the image quality evaluation of the gastroscope motion blurred images. This database is composed of 1,050 distorted images under five pixel levels for three different motion angles. It associates MOS values scored by 15 experienced and inexperienced viewers. What's more, we compared 20 FR IQA models by combining different features of images. The IFC, VIF, FSIM, and NQM achieved high consistency with the subjective scores. The results of the comparison show that visual saliency information, structure information, and image gradients are crucial features when devising objective IQA algorithms for gastroscope images. We then extracted and learned these features to design a novel IQA metric GIQE by adopting semi-full combination subspace. The results of the experiments imply that the proposed GIQE has always achieved a superior performance (i.e., better consistency) than the 20 existing FR IQA metrics. In the future, we would like to choose more lossless images to increase the capacity of the database. In addition, we would like to develop a higher performance objective IQA model for gastroscope images.

## Data availability statement

The original contributions presented in the study are included in the article/supplementary material, further inquiries can be directed to the corresponding author.

## Ethics statement

Written informed consent was obtained from the individual(s) for the publication of any potentially identifiable images or data included in this article.

## Author contributions

PY completed the first draft of the paper and confirmed the idea. RB completed the follow-up correction and modification of the paper. YY participated in the algorithm design of the paper. SL completed the experimental part of the paper. JW completed the summary part of the paper. CC completed the data collection part of the paper. QW completed the text correction and data collection part of the paper. All authors contributed to the article and approved the submitted version.
